# Development of a Multiplex Sandwich Aptamer Microarray for the Detection of VEGF_165_ and Thrombin

**DOI:** 10.3390/s131013425

**Published:** 2013-10-03

**Authors:** Alice Sosic, Anna Meneghello, Agnese Antognoli, Erica Cretaio, Barbara Gatto

**Affiliations:** 1 Dipartimento di Scienze del Farmaco, Università di Padova, via Marzolo 5, 35131 Padova, Italy; E-Mail: alice.sosic@studenti.unipd.it; 2 Veneto Nanotech S.C.p.A., Via S. Crispino 106, I -35129 Padova, Italy; E-Mails: anna.meneghello@venetonantoech.it (A.M.); agnese.antognoli@venetonanotech.it (A.A.); erica.cretaio@venetonanotech.it (E.C.)

**Keywords:** VEGF, thrombin, aptamers, sandwich aptamer-microarray, angiogenesis

## Abstract

In this work we have developed a multiplex microarray system capable of detecting VEGF_165_ and thrombin. We recently described a Sandwich Aptamer Microarray (SAM) for thrombin detection feasible for use in multiplex microarrays; here we describe a new aptasensor for VEGF_165_ detection employing Vap7 and VEa5, two DNA aptamers recognizing different sites of the protein. The aptamers were modified to be adapted to the solid phase platform of SAM and their capability to simultaneously recognize VEGF_165_ by forming a ternary complex was analyzed in solution. Having so defined the best tandem arrangement of modified aptamers, we set up the aptasensor for VEGF_165_, and finally analyzed the multiplex system with the two aptasensors for the simultaneous detection of VEGF_165_ and thrombin. The results indicate that each sandwich is specific, even when the two proteins are mixed. The system performance is consistent with the behavior evidenced by the biochemical analysis, which proves to be valuable to drive the evaluation and refinement of aptamers prior to or along the development of a detection platform. Since thrombin upregulates VEGF expression, the simultaneous recognition of these two proteins could be useful in the analysis of biomarkers in pathologies characterized by neo-angiogenesis.

## 1. Introduction

Aptamers are DNA or RNA molecules, obtained through evolutionary approaches, whose tridimensional conformation enables them to recognize a specific biomolecule with affinities comparable to those of antibodies [1]. Diagnostic approaches based on aptamers allow the realization of robust and reproducible systems devoid of the irregular functional performance and low storage stability typical of antibody-based systems. Aptamers in fact exhibit high performance consistency across different production batches, plus a wide range of temperature stability and flexibility towards various chemical modifications. For these reasons aptamers have found increasing applications in biosensor development for the recognition of proteins involved in pathological processes [2,3].

We recently set up a biosensor for human thrombin detection where we first examined in solution the effects of the post-SELEX modifications introduced in aptamers on the formation of binary and ternary complexes with the target. The biochemical characterization was followed by the design and achievement of a Sandwich Aptamer Microarray (SAM), using one DNA aptamer adapted to the immobilization on the surface of glass slides and a second aptamer modified for fluorescence detection [4,5]. The thrombin microarray resulted specific and efficient, therefore representing a potential tool for multiplex detection of disease-related biomarkers. Apart from its central role in blood coagulation, thrombin plays a role also in angiogenesis. The angiogenic action of thrombin has been demonstrated both *in vivo* and *in vitro* [6–8], and is mediated by the induction of expression of growth factors such as VEGF in tumor cells [6], retinal cells [9] and in human adipose tissue [10]. Angiogenesis is a complex and highly regulated event that consists in the sprouting of new capillaries from pre-existent vessels, a phenomenon strictly controlled by pro- and anti-angiogenic factors [11–13]. The control of such a diverse pattern of mediators can undergo deregulation resulting in the neovascularization that allows tumors to grow and metastasize [14,15]. Pathological angiogenesis also plays a crucial role in non-neoplastic diseases such as age-related retinopathies, leading to irreversible vision loss, and in chronic inflammatory disorders such as spondyloarthropathies (SpA) [11,16]. The vascular endothelial growth factors (VEGFs) are a major family of growth factors involved in these pathogenic processes. Five VEGF isoforms are generated as a result of alternative splicing from a single VEGF gene, differing in their molecular mass and in biological properties [17]. VEGF_165_ is the most abundant splice variant of VEGF-A and its presence is widely correlated in the literature to pathological occurrence and neoplastic progression, with highly variable protein levels as determined by sandwich-based immunoassays employing anti-VEGF antibodies [18].

Given the relevance of angiogenesis in physio-pathological processes, the set-up of biosensors for angiogenesis-related diseases is urgently needed. VEGF_165_ is a homodimeric glycoprotein consisting of two domains: a heparin-binding domain (HBD) [19,20] and a receptor-binding domain (RBD) [21,22], thus representing an optimal target for the development of an aptasensor in a sandwich format. Recently, different research groups have isolated DNA aptamers able to bind VEGF: Hasegawa and collaborators characterized two related DNA aptamers (VEa4 and VEa5) recognizing all VEGFs presenting an HBD [23]. VEa5 in particular binds VEGF_165_ with a *K_d_* value of 130 nM [23]. In a recent work it was demonstrated that VEa5 aptamer binds VEGF_165_ isoform in colorectal cancer cells [24]. Nonaka and collaborators identified another DNA aptamer, named Vap7, that recognizes the RBD of VEGF_165_ with a *K_d_* value of 20 nM [25]. Vap7 is the first VEGF_165_ binding aptamer that folds into G-quadruplex structure [25], while the three dimensional folding of VEa5 is not known: its secondary structure, predicted using the Zuker DNA folding algorithm, is supposed to involve three stem-loops [23].

Based on reported data on these aptamers and following the experimental approach used in our previous works on thrombin [4,5], we employed Vap7 and VEa5 to develop a Sandwich Aptamer Microarray (SAM) for VEGF_165_. No reports are published, at the best of our knowledge, about the adaptability of Vap7 and VEa5 as capture or detection layer for a VEGF aptasensor design. Previous to the SAM development, we therefore analyzed how post-SELEX chemical modifications introduced in these aptamers would affect VEGF_165_ recognition in solution and identified the optimal arrangement of modified Vap7 and VEa5. By Electrophoretic Mobility Shift Assay (EMSA) we demonstrated that the ternary complex can be obtained with higher efficiency using a specific tandem set of the modified aptamers, although in all cases with loss of affinity to VEGF_165_. Using the information from the biochemical analysis, the system was applied to the solid phase for VEGF-capture. After the set-up of the VEGF_165_ aptasensor, we built a multiplex SAM coupling the new VEGF_165_ aptasensor to the thrombin one, demonstrating the specificity of recognition and the possibility of simultaneous detection of the two proteins. The multiplex microarray employing specific aptamers in the sandwich format is suitable for further development: addition of new aptasensors for the detection of validated biomarkers could allow applications in several pathologies ranging from tumorigenesis and metastasis to atherosclerosis and metabolic syndrome.

## 2. Experimental Section

### 2.1. Aptamers and Proteins

All DNA oligonucleotides were purchased from IDT Integrated DNA Technologies (Leuven, Belgium). The DNA sequences of the unmodified Vap7 and VEa5 aptamer against VEGF_165_ were, respectively, 5′-ATA CCA GTC TAT TCA ATT GCA CTC TGT GGG GGT GGA CGG GCC GGG TAG A-3′ and 5′-ATA CCA GTC TAT TCA ATT GGG CCC GTC CGT ATG GTG GGT GTG CTG GCC AGA TAG TAT GTG CAA TCA-3′. Both the 49-mer Vap7 aptamer and the 66-mer VEa5 aptamer were synthetized with a 5′-amino modification (Vap7-NH_2_ and VEa5-NH_2_). Vap7 was also synthetized with a 5′-amino modification plus a polyT(12) tail as spacer (reported as Vap7(12T)NH_2_). Both Vap7 and VEa5 were used as 5′-FAM labeled (Vap7-FAM and VEa5-FAM) and VEa5 aptamer was used also as 5′-Cy5 labeled (VEa5-Cy5). In the Sandwich Aptamer Microarray (SAM) an unrelated 5′-NH_2_ modified DNA oligonucleotide (Random DNA: 5′-TCA CGC TTA TTT AAG AAG CTG TTT GGT GGA GG-3′) was used as negative control. As reported in our previous works [4,5], the sequence of the unmodified 15-mer TBA1, used as selection aptamer, is: 5′-GGT TGG TGT GGT TGG-3′. To allow immobilization on microarray slides, an amino modification plus a polyT(12) spacer were added at the 5′ terminus [TBA1(12T)NH_2_]. The sequence of the unmodified 29-mer TBA2 is: 5′-AGT CCG TGG TAG GGC AGG TTG GGG TGA CT-3′. TBA2 was used as detection aptamer with a 5′-Cy5 modification (TBA2-Cy5).

Human recombinant Vascular Endothelial Growth Factor 165 (VEGF_165_) and Human α-thrombin were purchased from Sigma-Aldrich (St. Louis, MO, USA), resuspended, aliquoted and stored as recommended by the supplier.

### 2.2. EMSA (Electrophoresis Mobility Shift Assay) Analysis for Binary and Ternary Complexes

The binding of each aptamer to the protein target in solution was analyzed by EMSA. Prior to incubation with VEGF_165_, all the aptamer samples were folded: unmodified and modified VEa5 aptamers were folded in TBSE buffer (0.05 mM EDTA, 10 mM Tris-HCl, 100mM NaCl, pH 7), while to fold unmodified and modified Vap7 aptamers 100 mM KCl was added to the TBSE buffer. 1 μM Vap7 aptamers in the presence of TBSE + KCl buffer (0.05 mM EDTA, 10 mM Tris-HCl, 100 mM NaCl, 100 mM KCl, pH 7), or 1 μM VEa5 aptamers in TBSE buffer were denatured at 95 °C for 5 min and the samples were left to cool down to room temperature (slow annealing). Each folded aptamer (0.1 μM) was then incubated with increasing concentrations (from 0 to 10 μM) of VEGF_165_ in a total volume of 10 μL, at 25 °C for one hour. For ternary complex, in order to verify the sandwich formation in solution, a Supershift Assay was performed and two possible combinations of aptamers were tested: Vap7-NH_2_ as capture layer and VEa5-FAM as detection layer; VEa5-NH_2_ as capture aptamer and Vap7-FAM as detection aptamer. Both pairs of folded aptamers (0.1 μM each) were incubated simultaneously with the protein (5 and 10 μM) in TBSE buffer in a total volume of 10 μL, at 25 °C for 30 min. After incubation, free aptamer and VEGF_165_-aptamers complexes were resolved by 12% non-denaturing polyacrylamide gels containing glycine buffer (glycine 50 mM pH 9.5) and 10 mM KCl (Vap7 aptamers) or glycine buffer only (VEa5 aptamers). Due to high VEGF_165_ pI [26], a 50 mM glycine buffer at pH 9.5 (with or without KCl) was used as buffer in the gels and as running buffer for EMSA in order to have the protein negatively charged. To visualize oligonucleotides in gel systems the fluorescence of Vap7-FAM and VEa5-FAM was directly detected on a Geliance 600 Imaging System (PerkinElmer, Waltham, MA, USA).

### 2.3. Aptamer Preparation for SAM and Printing of Aptamer Microarrays

In the aptamer arrays Vap7 was used as capture layer for VEGF_165_. Negative control DNA and Vap7 were anchored on distinct spots on a microarray slide. To improve capture-protein efficiency, Vap7 was modified also with a 5′- polyT (12T) spacer. In this way the G-quadruplex structure can fold correctly to recognize and efficiently bind the target protein. Prior to immobilization, Vap7(12T)NH_2_ (80 μM in TBSE buffer and KCl 100 mM) was denatured at 95 °C for 5 min and then left to cool down to room temperature. Folded aptamer and Random DNA (control sequence) were diluted in Microarray Printing Buffer 1.5X (Printing Buffer 6 ×: 300 mM sodium phosphate, 0.02% Triton, pH 8.5) to a final concentration of 20 μM. These probes were loaded into micro-plates and submitted to the Spotter Arrayer (Versarray Chip Writer Pro System, Bio-Rad Laboratories Pty, Ltd, Hercules, California, USA) for slide printing.

E-surf LifeLine slides (25 mm × 75 mm, LifeLineLab, Pomezia, Italy) were used since they allow the binding of amino-modified DNA sequences. Slides were printed by Spotter Arrayer instrument, using Telechem SMP3 microspotting pins. Printed slides were incubated overnight in a 75% humidity incubation chamber, blocked at 30 °C in Microarray Blocking Solution (0.1 M Tris, 50 mM ethanolamine, pH 9), washed at 30 °C in Microarray Washing Solution (4× SSC, 0.1% SDS) and finally washed in MilliQ H_2_O, spin-dried and stored properly until usage. Each slide has the possibility to test up to 12 or 48 samples at once, since each sub-array can be physically isolated from the others by the Corning Microarray Hybridization Chamber (Sigma-Aldrich, St. Louis, MO, USA). This approach ensures to incubate up to 48 samples at the same time and nevertheless to minimize array variation resulting from minor fluctuation of external parameters.

### 2.4. Proteins Labeling

The AlexaFluor^®^555 monoreactive Succinimidyl Esters (Invitrogen, Carlsbad, CA, USA), dissolved in anhydrous dimethyl sulfoxide (DMSO) was used to label VEGF_165_ samples. Prior to labeling, the protein and AlexaFluor^®^ 555 were first diluted to proper reaction concentrations in MilliQ H_2_O. The labeling reaction was done mixing VEGF_165_ samples with the AlexaFluor^®^555 NHS esters reagent in the Protein Coupling Buffer (0.1 M sodium carbonate, pH 9.4); in this case, the optimal dye to protein ratio was 1. The reaction was incubated 2 h at 4 °C, and all the free unreacted AlexaFluor^®^555 NHS esters were finally blocked with an excess of glycine. The same protocol was used also to label thrombin with AlexaFluor^®^647. The labeled protein samples were kept in the dark at 4 °C, and all samples were used within a week.

### 2.5. Detection Layer Preparation

The VEa5-Cy5 aptamer was used as detection aptamer against human VEGF_165_: VEa5-Cy5 (10 μM in TBSE buffer) was denatured at 95 °C for 5 min and then left to cool down to room temperature in order to assume the correct structure for recognition of the heparin binding domain (HBD) of VEGF_165_. The TBA2-Cy5 aptamer was used to detect human thrombin as already reported [4,5].

### 2.6. Sandwich Aptamer Microarray Assays

The printed aptamer microarrays prepared as detailed above were immersed in TBSE containing 100 mM KCl at room temperature for 15 min, just before usage.

The Sandwich Aptamer Microarrays for VEGF_165_ were performed with two procedures, *i.e.*, analyzing the sandwich formation on surface either in a “one-step” or in a “two-steps” protocol. The “one-step” procedure consisted in the pre-incubation of protein with the fluorescently labeled aptamer (VEa5-Cy5) in solution in TBSE as binding buffer, at 25 °C for 30 min. The pre-formed complex thus obtained was then incubated on the microarray at 25 °C for 30 min. The “two-steps” procedure consists instead in the incubation on the microarray of VEGF_165_ diluted in TBSE at 25 °C for 30 min, followed by the incubation with the fluorescently labeled secondary aptamer (VEa5-Cy5) diluted in TBSE, at 25 °C for further 30 min. Finally, the aptamer microarrays were carefully rinsed with PBS (10 mM phosphate, 137 mM NaCl, 3 mM KCl, pH 7.4), washed three times with PBS at room temperature to remove the unbound proteins, rapidly rinsed in MilliQ H_2_O and spin-dryed.

### 2.7. Multiplex Sandwich Aptamer Microarray Assays

**Figure 1.**
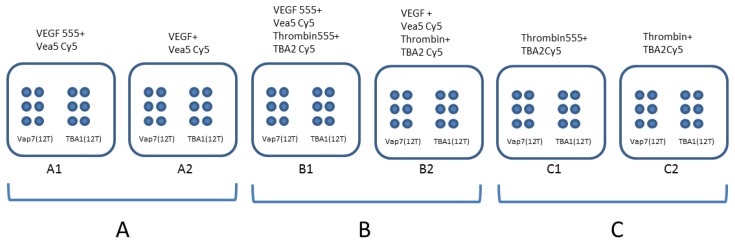
Scheme of the microarray printed glass slide. A1: detection of VEGF555; A2: detection of VEGF. B1: detection of VEGF555 mixed with thrombin555; B2: detection of VEGF mixed with thrombin. C1: detection of thrombin555; C2: detection of thrombin.

For the experiment also involving thrombin, detection layer, capture layer and fluorescent thrombin were prepared and incubated as previously described [5]. Different proteins amounts were incubated in presence of detection pre-folded aptamers (VEa5-Cy5 and TBA2-Cy5) using the “one-step” procedure. The experiments were conducted using each protein (VEGF_165_ and thrombin) alone or mixed together, as shown schematically in Figure 1. The binary complexes were analyzed on slides printed with both Vap7(12T)NH_2_ and TBA1(12T)NH_2_. The slide contains 6 identical sub-array, each of them with six spots of Vap7(12T)NH_2_ aptamer and six of TBA1(12T)NH_2_ aptamer. In Panel A the AlexaFluor^®^ 555 labeled (VEGF555) or unlabeled VEGF_165_ protein (VEGF) bound to VEa5-Cy5 aptamer was incubated on both sub-arrays. The same experiment was conducted with human thrombin (labeled and unlabeled) on Panel C and in presence of the mixed VEGF and thrombin (labeled and unlabeled) in Panel B.

### 2.8. Slides Scanning and Data Analysis

Spin-dried aptamer arrays were scanned using Genepix 4000B laser scanner (Molecular Devices, LLC, Sunnyvale, CA, USA) and the Gene Pix Pro software using both 532 nm and 635 nm wavelength. Fluorescent spot intensities were quantified using the Gene Pix Pro software after normalizing the data by subtracting local background from the recorded spot intensities.

## 3. Results and Discussion

### 3.1. VEGF_165_ Recognition by Post-SELEX Modified Vap7 and VEa5

Since Vap7 and VEa5 aptamers can bind VEGF_165_ to two different epitopes, namely the RBD and the HBD respectively, we planned to use them in tandem for the construction of a VEGF_165_ Sandwich Aptamer Microarray (SAM). Using the same approach adopted for the development of the thrombin aptasensor [5], an extensive analysis in solution on the aptamer-protein complexes formation was performed prior to the development of the array system. We investigated by EMSA the effects on aptamers-VEGF_165_ binding of an amino group addition, necessary for surface immobilization, and of a fluorescent reporter conjugation. We verified that the VEGF_165_ recognition by unmodified aptamers was maintained in the experimental conditions (data not shown), and proceeded to analyze the effects of the chemical modifications on binary and ternary complexes with the protein.

**Figure 2.**
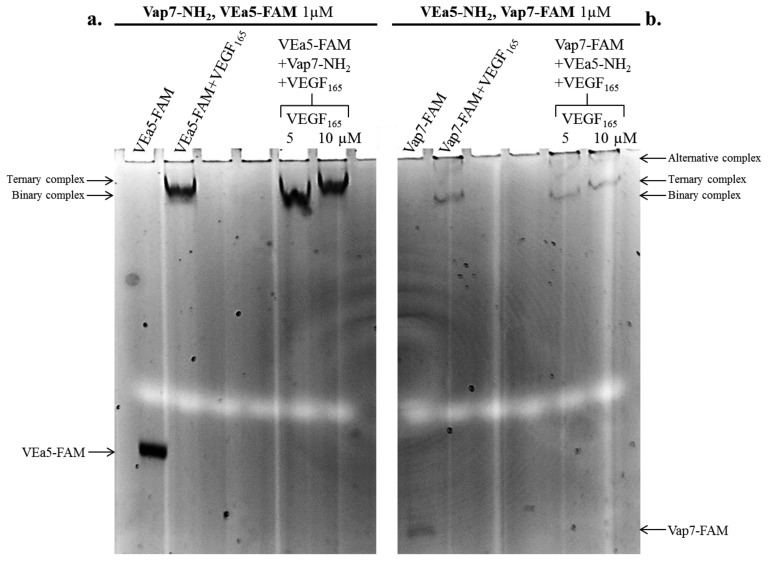
Electrophoretic Mobility Supershift Assay of Vap7-NH_2_ + VEGF_165_ + VEa5-FAM (**a**) and of VEa5-NH_2_ + VEGF_165_ + Vap7-FAM (**b**). In Panel (a) the Vap7-NH_2_-VEGF_165_-VEa5-FAM interaction was analyzed, while in Panel (b) the VEa5-NH_2_-VEGF_165_-Vap7-FAM interaction was analyzed. Each aptamer was incubated separately or simultaneously with the protein under the described conditions. The respective aptamer and VEGF_165_ concentrations are indicated in the figure. Binding reactions were applied on a 12% non-denaturing PAA gel containing glycine buffer and 10 mM KCl. The mobility of free and complexed aptamers, detected by the FAM fluorescence, was analyzed using the Geliance 600 Imaging System.

The formation of the ternary complex was analyzed with two alternative combinations of aptamers: (a) Vap7-NH_2_ as capture aptamer plus VEa5-FAM as detection aptamer are shown in Figure 2a (left lanes in the gel); (b) the arrangement with VEa5-NH_2_ as capture layer and Vap7-FAM as detection layer is shown in Figure 2b (lanes on the right). Free aptamers and VEGF_165_-aptamer complexes were prepared and resolved by 12% non-denaturing polyacrylamide gel, as reported in the Experimental Section. Free aptamers and the binary complex of each aptamer with VEGF_165_ were loaded in the gel as controls. The detection of the FAM fluorescence signal allows to identify the shift of the fluorescent aptamer (VEa5-FAM in Panel **a**, or Vap7-FAM in Panel **b**) and evidences binding to the protein.

In Figure 2a (Vap7-NH_2_ and VEa5-FAM) the free fluorescent aptamer is strongly shifted up in the gel in the presence of 5 µM VEGF_165_ (binary VEa5-FAM-VEGF_165_ complex). When VEa5-FAM and Vap7-NH_2_ were incubated simultaneously with 10 µM VEGF_165_ the fluorescent band is super-shifted, exhibiting a lower electrophoretic mobility than the binary complex, consistent with the formation of the ternary complex. The biochemical analysis indicates however that the ternary complex with VEGF_165_ is weaker than expected, since it is evident only at the higher concentrations of protein: at 5 µM protein no ternary complex formation is evident in solution, indicating that the aptamer sandwich has lower affinity for VEGF_165_ at the basic pH conditions employed.

The alternative tandem of modified aptamers (VEa5-NH_2_ + Vap7-FAM) was also investigated (Figure 2b): the binary complex formation is confirmed by the shift of the free Vap7-FAM in the presence of VEGF_165_ 5µM and the ternary complex is evidenced by the supershift of Vap7-FAM in the simultaneous presence of VEa5-NH_2_ and 10µM VEGF_165_. Two bands with different electrophoretic mobility appear when the fluorescent Vap7 binds the protein, indicating the coexistence of two stable alternative complexes. In addition, it is immediately evident, comparing Panel b to Panel a, that the reporter′s fluorescence is quenched when the fluorophore is conjugated to Vap7 aptamer (free Vap7-FAM) and becomes even weaker when Vap7-FAM interacts with the target protein. Since this quenching effect could interfere with the sandwich detection in solid phase, our preliminary biochemical analysis indicates that, in solution, the first arrangement analyzed, Vap7-NH_2_ - VEGF_165_ - VEa5-FAM, appears to be more suitable for the SAM development. Therefore, in our preferred solid phase setting Vap7-NH_2_ will be used as capture layer while the fluorescently labeled VEa5 will be used as detection layer.

### 3.2. Development of the SAM for VEGF_165_

The sandwich system with the post-SELEX modified aptamers analyzed in solution was then applied to the solid phase. The first step of the analysis was to verify the VEGF_165_ recognition by the immobilized capture aptamer: Vap7 is a long sequence (49 nucleotides), but the accessibility of the G-quadruplex motif of the immobilized aptamer to VEGF_165_ may be hindered in solid phase. A polyT spacer of 12 thymidines was therefore added to the 5′-end, similarly to what performed in the development of the thrombin sensor [5]. Employing Alexa555-labeled VEGF_165_ we evaluated the interaction of the protein with the two immobilized aptamers, demonstrating that Vap7(12T)NH_2_ anchored on the slides gave a better defined and sharper signal when the glass slide was incubated with Alexa555-labeled VEGF_165_. Vap7(12T)NH_2_ aptamer was therefore used for the development of the SAM for VEGF_165_.The results of this experiment are reported in Supplementary Figure 1.

As detection aptamer, the fluorescent 5′-Cy5 modified VEa5 (VEa5-Cy5) was used and the sandwich formation was detected by its red fluorescence. Alexa555-labeled VEGF_165_ was also used as control to further verify the sandwich formation by the yellow fluorescence resulting from the co-localization of green (Alexa-conjugated protein) and red (Cy5-conjugated aptamer) fluorophores. We tested and compared two different protocols (“one-step” and “two-steps”), as previously done for the thrombin sensor [5]. The results of the experiments, reported in Supplementary Figure 2, evidence that the sandwich was specifically formed for the VEGF_165_ aptamer while no signal was detected for the negative control. SAM was obtained with VEGF_165_ and also with Alexa-labeled VEGF_165_, and the “one-step” protocol was shown to be more efficient than the “two-steps” procedure. We can conclude that the SAM protocol employing the devised tandem arrangement of these aptamers is successful for the detection of VEGF_165_.

### 3.3. Specificity of Aptamer Recognition

**Figure 3.**
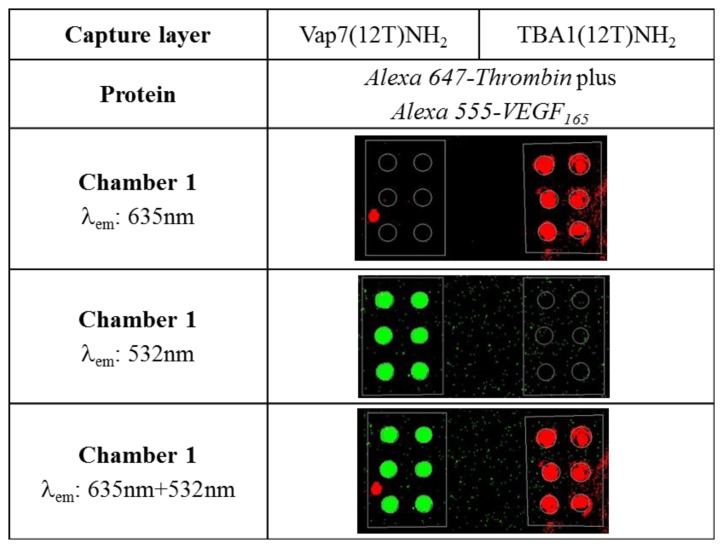
Images of the microarray slide after the simultaneous incubation of Alexa555-VEGF_165_ and Alexa647-thrombin (each 500 nM). On the same cell, Vap7(12T)NH_2_ was anchored as capture layer for VEGF_165_ (left) and TBA1(12T)NH_2_ was anchored as capture layer for thrombin (right). Green fluorescence (λ^em^: 532 nm) represents labeled VEGF_165_ (Alexa 555) and red fluorescence (λ^em^: 635 nm) represents labeled thrombin (Alexa 647).

We already demonstrated that VEGF_165_ is not recognized by the aptamers used in the SAM for thrombin [4] despite the fact that the two proteins are structurally characterized by similar domains [27]. Considering the possible applications of the two systems in multiplex microarray detection in which the two proteins are mixed together, we verified the specificity of protein capture and the possible interference on the recognition of the immobilized aptamer. Alexa555-VEGF_165_ and Alexa647-thrombin were mixed and incubated simultaneously in a chamber in which Vap7(12T)NH_2_ (for VEGF_165_) and TBA1(12T)NH_2_ (for thrombin) were printed as capture layers in the same sub-array chamber. Recognition of VEGF is evidenced by the green fluorescence while the red fluorescence of Alexa647 witnesses the capture of thrombin. The microarray scan obtained reading the two different wavelengths is shown in Figure 3.

The experiment reported above shows that both proteins, mixed and applied to the glass slide at the same time, do not interfere with each other for the recognition of the immobilized aptamer. The specificity of recognition is evident by the reading of the two fluorescence performed simultaneously, also evidencing a low background by Alexa555-VEGF.

### 3.4. Development of the Multiplex SAM for VEGF_165_ and Thrombin

**Figure 4.**
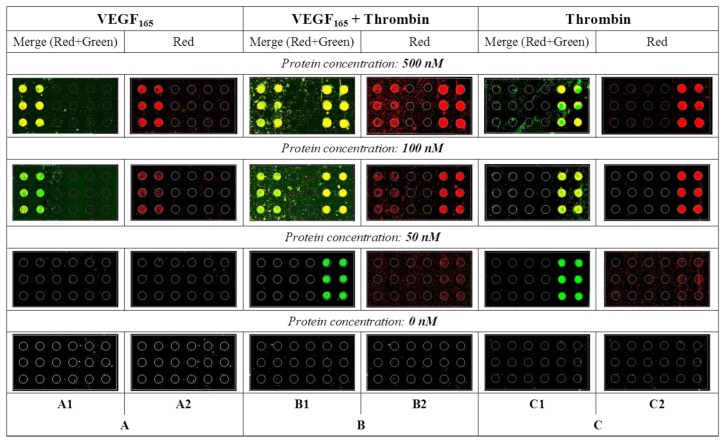
Images of the microarray slides reporting the multiplex SAM for VEGF_165_ and thrombin detection. The six spots printed with Vap7(12T)NH_2_ are on the left of each subarray and the six spots printed with TBA1(12T)NH_2_ on the right. Panel A reports the detection of human VEGF_165_, Panel C the detection of human thrombin. Panel B reports the simultaneous detection by each aptasensor of the specific target mixed in solution. Each protein, at the same concentration, was then incubated separately with its own specific detection aptamer (VEa5-Cy5 or TBA2-Cy5) and analyzed on the glass slide. Cy-5 detection aptamers, namely VEa5-Cy5 for VEGF_165_ or TBA2-Cy5 for thrombin, were 1 μM. Glass slides A1, B1 and C1 were incubated with Alexa-555 labeled proteins, while slides A2, B2 and C2 were incubated with unlabeled proteins.

Having established that each protein, is also specifically recognized and fixed on the glass slide in a complex mix by its proper capture aptamer, we proceeded with the development of multiplex SAM for the simultaneous detection of mixed VEGF_165_ and thrombin. Figure 4 reports the results of these experiments performed at different protein concentrations.

The protocols adopted for each detection were the same and each glass slide was printed in the same way: six spots of Vap7(12T)NH_2_ on the left and six spots of TBA1(12T)NH_2_ on the right. Each protein, at the same concentration, was then incubated with its own specific detection aptamer (VEa5-Cy5 for VEGF_165_ or TBA2-Cy5 for thrombin) and analyzed on the glass slide. We evaluated the two sandwiches side by side, first with each protein alone (Figure 4, Panels A and C) and then with the two proteins mixed (Figure 4, Panel B). Red signals of Panels A2, B2 and C2 correspond to the detection of Cy5-conjugated aptamer bound to its protein, while the yellow signal results from the merge of the green fluorescence of Alexa555-conjugated protein with the red signal of Cy5-conjugated detection aptamers (Panels A1, B1 and C1). The central Panel of Figure 4 (Panel B: Alexa555-labeled proteins in Panel B1 or unlabeled proteins in Panel B2) reports the results obtained when the two proteins are mixed in solution, incubated with the two Cy-5 labeled detection aptamers and then applied to the glass slides printed with the specific anchored capture aptamers.

No unspecific signals are observed when the two proteins were analyzed alone (Panels A and C): each SAM captures and reveals specifically the presence of its target protein. The results of the experiments performed with mixed proteins is striking: the VEa5 aptamer, recognizing the HBD domain of VEGF, does not impair the recognition of thrombin HBD by the immobilized TBA1 that we employed as capture aptamer in the SAM for thrombin [5]. Each aptasensor is specific and they can be used to detect their mixed target. Our experiment suggests that the SAM system could be exploited for the development of a multiplex microarray for protein analysis in the presence of a single detection strategy. However, it is immediately evident that the sensitivity of each separate system is different, since the SAM for thrombin detects even the lowest protein concentrations used, while the SAM for VEGF_165_ is less sensitive, with the signal hardly detectable below 50 nM. The low sensitivity of the VEGF_165_ aptasensor is in agreement with what we have evidenced during the analysis of ternary complex formation in solution, and also consistent with the higher dissociation constants of the VEGF_165_ aptamers (Vap7 *K_d_* = 20 nM, VEa5 *K_d_* = 130 nM) [23,25] compared to those reported for thrombin aptamers (TBA1 *K_d_* = 26 nM, TBA2 *K_d_* = 0.5 nM) [28,29]. Clearly, to employ the VEGF_165_ DNA aptasensor for quantitative detection, modified or biased re-selected aptamers should be optimized for the SAM, hopefully without losing the specificity to the target that we have here demonstrated.

## 4. Conclusions

VEGF_165_ aptamers, besides representing an important achievement in the field of therapeutic aptamers, are feasible to be employed in a variety of diagnostic applications. In the context of our interest for aptasensors arrays for relevant biomarkers detection, the simultaneous detection of thrombin and VEGF_165_ is particularly interesting: thrombin stimulates dose dependently VEGF_165_ in retinal pigmented cells (RPE) up to 4 ng/mL [9], promoting angiogenesis. Thrombins-induced VEGF_165_ expression in inflammation linked to obesity has also been analyzed: higher levels of VEGF (over 13 ng/mL) were measured in preadipocytes upon thrombin stimulation, highligthing the association of thrombin with neoangiogenesis in atherosclerosis and metabolic syndrome [10]. The serum concentration range of VEGF_165_ in healthy individuals, as reported by ELISA kits producers, is below 1ng/mL, both for males and females, while the plasmatic concentration is not higher than 100 pg/mL. In pathological conditions, the range of VEGF_165_ concentration is therefore clearly higher and non-overlapping with the normal one. Thrombin-induced tumorigenesis and metastasis is associated with enhanced VEGF_165_ protein synthesis and secretion [6,30], although with high variability in VEGF levels reported by several authors [31,32].

With a view to the development of a multiplex microarray system for the detection of these two proteins we set up a SAM for the analysis of VEGF_165_ employing two DNA aptamers described in literature and reported to bind different and mutually exclusive target domains. Few data concerning Vap7 and VEa5 aptamers and their interaction with VEGF_165_ are described. Therefore, we introduced minimal changes to the published sequences to adapt them to their function in the SAM, *i.e.*, protein capture and protein detection, taking advantage of the chemical modifications demonstrated successfully for thrombin. Analysis and optimization of binary and ternary complexes formation between the modified anti-VEGF aptamers and their target in solution were performed to verify the sandwich formation. The solution experiments point to the requirement of careful handling of anti-VEGF aptamers when adapting their sequences to the solid phase: the presence of a labeling dye conjugated to both the Vap7 and the VEa5 aptamers allows the protein recognition, since the binary complex formation is not abolished, but it induces a decrease in binding affinity.

In solid phase the G-quadruplex motif of Vap7 was not completely accessible to the protein, and a spacer was added to obtain Vap7(12T)NH_2_, anchored on the glass slides as capture layer. With these modifications the SAM proved successful alone and also in the multiplex setup: the simultaneous detection of VEGF_165_ and thrombin by each SAM proved to be specific, and no cross-recognition was observed. Our results demonstrate that both VEGF_165_ aptamers are able to recognize their specific target, even in the presence of thrombin. However, although the correct sandwich aptamer formation was verified, the performance of VEGF_165_ aptasensor is not comparable to that of thrombin. The lower detection of VEGF_165_, compared to that of thrombin, is in agreement with the reported *K_d_* of the VEGF-aptamers, higher than those reported for thrombin. Thrombin aptamers are short and very compact compared to the longer VEGF aptamers, and have been extensively used by several authors for aptasensor development based on different technologies. VEGF aptamers are on the opposite not so well characterized and, at the best of our knowledge, never employed for detection in a sandwich system. No structural characterization of the G-quartet arrangement of Vap7 is known, while VEa5 structure has been recently optimized for the recognition of the HBD of VEGF_165_: an SPR analysis showed that the truncation of the VEa5 sequence leads to an improvement of protein recognition [24]. This report indicates that refinement of VEGF aptamers is needed to develop aptasensors with performances comparable to the reported systems based on antibodies (ELISA) [18], RNA aptamers/antibodies coupled to amplification methods [33], or a very sensitive nanoplasmonic aptasensor using a high affinity RNA aptamer [34].

We believe that the results presented here strongly indicate that a multiplex approach to the development of aptasensors for related biomarkers involved in pathological angiogenesis could be pursued. Aptasensors development should be guided by a thorough biochemical analysis, substantiating our approach of verifying in solution the effects of aptamers modifications prior or along the development of the solid phase. To the best of our knowledge this is the first example showing that two VEGF aptamers, binding different epitopes of the same target, are feasible to the design and development of an aptasensor in the same format adopted by the commercially available assays, which are based on anti-VEGF antibodies. Despite the advantages and the similarities in assay design, aptasensors are yet not as popular as antibody-based sensors. We hope that our work will contribute to pursue SELEX as a mean to rapidly identify “couples” of non-overlapping aptamers, *i.e.*, binding to different epitopes of the same target, for their application in tandem in all those diagnostic formats now extensively exploited by antibodies.

## Supplementary Material
