# Supplementary material for: Development of a Multiplex Sandwich Aptamer Microarray for the Detection of VEGF165 and Thrombin
Source: Sensors (Basel). 2013 Oct 3;13(10):13425–38. doi: 10.3390/s131013425 (PMC3859071; doi:10.3390/s131013425)
Supplement: Supplementary file 1 [file sensors-13-13425-s001.pdf]

OPEN ACCESS
*sensors*
**ISSN 1424-8220**
www.mdpi.com/journal/sensors

*Supplementary Information*

# Development of a Multiplex Sandwich Aptamer Microarray for the Detection of $VEGF_{165}$ and Thrombin

**Alice Sosic [1], Anna Meneghello [2], Agnese Antognoli [2], Erica Cretaio [2] and Barbara Gatto [1,*]**

[1] Dipartimento di Scienze del Farmaco, Università di Padova, via Marzolo 5, 35131 Padova, Italy; E-Mail: alice.sosic@studenti.unipd.it
[2] Veneto Nanotech S.C.p.A., Via S. Crispino 106, I -35129 Padova, Italy; E-Mails: anna.meneghello@venetonantoech.it (A.M.); agnese.antognoli@venetonanotech.it (A.A.); erica.cretaio@venetonanotech.it (E.C.)

* Author to whom correspondence should be addressed; E-Mail: barbara.gatto@unipd.it; Tel.: +39-049-827-5717; Fax: +39-049-827-5366.

---

**Figure S1.** Images of a microarray slide after the incubation of Alexa555-labeled $VEGF_{165}$. Vap7(12T)$NH_2$ and Vap7-$NH_2$ were anchored on the glass slide as capture layer on the left and on the right, respectively. The green fluorescence (due to Alexa555 fluorophore) represents the bound labeled protein.

| **Capture layer** | Vap7 (12T)$NH_2$ | Vap7 $NH_2$ |
|---|---|---|
| **Chamber** | 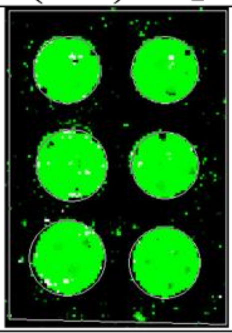 | 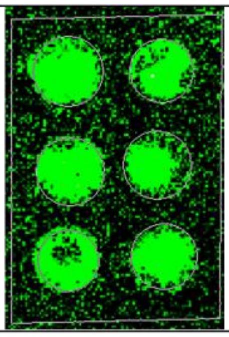 |
| **Protein** | Alexa555-labeled VEGF $_{165}$ | |

**Table S1.** Schematic representation of the SAM protocol for VEGF detection.

| Sub-array | 1 | | 2 | | 3 | | 4 | |
|---|---|---|---|---|---|---|---|---|
| **Capture layer** | Vap7 (12T)$NH_2$ | Random DNA-$NH_2$ | Vap7 (12T)$NH_2$ | Random DNA-$NH_2$ | Vap7 (12T)$NH_2$ | Random DNA-$NH_2$ | Vap7 (12T)$NH_2$ | Random DNA-$NH_2$ |
| **Protein** | $VEGF_{165}$ (0,1µM) pre-complexed with VEa5-Cy5(0,5µM) | | Alexa555-$VEGF_{165}$ (0,1µM) pre-complexed with VEa5-Cy5(0,5µM) | | $VEGF_{165}$ (0,1µM) | | Alexa555-$VEGF_{165}$ (0,1µM) | |
| **Detection layer** | | | | | VEa5-Cy5(0,5µM) | | VEa5-Cy5(0,5µM) | |
| **Procedure** | One-step | | | | Two-steps | | | |

**Figure S2.** Images of the microarray slide for the Sandwich Aptamer Microarray (SAM) for VEGF. In each subarray, Vap7(12T)$NH_2$ was printed on the left while the negative control (Random DNA-$NH_2$) was printed on the right. Red fluorescence represents binding by VEa5-Cy5. In chambers 2 and 4, in which Alexa 555-labeled $VEGF_{165}$ (500 nM) was incubated, the fluorescence of the yellow spots indicates the simultaneous co-localization of the protein (green) and of the detection aptamer (red).

| One-step protocol | | | | Two-steps protocol | | | | |
|---|---|---|---|---|---|---|---|---|
| 1 | | 2 | | 3 | | 4 | | |
| Vap7 (12T)$NH_2$ | Random DNA-$NH_2$ | Vap7 (12T)$NH_2$ | Random DNA-$NH_2$ | Vap7 (12T)$NH_2$ | Random DNA-$NH_2$ | Vap7 (12T)$NH_2$ | Random DNA-$NH_2$ | |
| 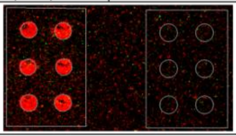 | | | | 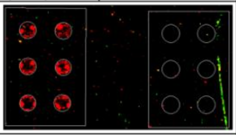 | | | | **Red fluorescence (Cy5)** |
| | | 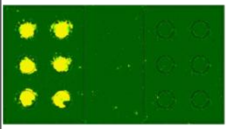 | | | | 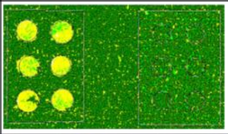 | | **Green and red fluorescence merge (Alexa 555 + Cy5)** |

© 2013 by the authors; licensee MDPI, Basel, Switzerland. This article is an open access article distributed under the terms and conditions of the Creative Commons Attribution license (http://creativecommons.org/licenses/by/3.0/).
